# 非小细胞肺癌不同部位组织样本PD-L1表达水平比较研究

**DOI:** 10.3779/j.issn.1009-3419.2022.102.14

**Published:** 2022-05-20

**Authors:** 小征 黄, 江华 吴, 立新 周, 志杰 宋, 婉彤 徐, 玲 贾, 新婷 刁, 琪 吴, 冬梅 林

**Affiliations:** 1 100142 北京，北京大学肿瘤医院暨北京市肿瘤防治研究所病理科，恶性肿瘤发病机制及转化研究教育部重点实验室 Department of Pathology, Peking University Cancer Hospital & Institute; Key Laboratory of Carcinogenesis and Translational Research (Ministry of Education), Beijing 100142, China; 2 300060 天津，天津医科大学肿瘤医院病理科，国家肿瘤临床医学研究中心，天津市“肿瘤防治”重点实验室，天津市恶性肿瘤临床医学研究中心 Department of Pathology, Tianjin Medical University Cancer Institute and Hospital; National Clinical Research Center of Cancer; Key Laboratory of Cancer Prevention and Therapy; Tianjin's Clinical Research Center of Cancer, Tianjin 300060, China

**Keywords:** 肺肿瘤, 程序性死亡配体1, 异质性, 肿瘤阳性比例评分, Lung neoplasms, Programmed cell death ligand 1, Heterogeneity, Tumor proportion score

## Abstract

**背景与目的:**

程序性死亡配体1（programmed cell death ligand 1, PD-L1）作为非小细胞肺癌（non-small cell lung cancer, NSCLC）免疫治疗患者分层标志物已进入临床病理常规检测。然而PD-L1表达在肺内和肺外不同转移部位的空间异质性是困扰临床检测的难题。本研究旨在探讨NSCLC不同部位组织样本的PD-L1表达评分的差异，从而有助于晚期肺癌患者的PD-L1检测策略的制定。

**方法:**

回顾性收集PD-L1（22c3抗体，Dako）临床病理连续检测的131例肺外转移性NSCLC以及同期非配对肺内肿瘤972例进行对照分析，对比肺外与肺内肿瘤组织样本检测的PD-L1肿瘤阳性比例评分（tumor proportion score, TPS）差异。

**结果:**

肺外转移性NSCLC的PD-L1阳性表达率（TPS≥1%）为61.83%，TPS评分显著高于同期肺内肿瘤（*P*=0.03）。不同部位组织样本的PD-L1表达评分具有显著差异（*P*=0.007）。肝脏和肾上腺转移瘤的PD-L1阳性率高，分别为85.71%和77.78%，其TPS评分均显著高于肺内肿瘤（*P* < 0.05）。淋巴结、骨、脑、软组织和胸膜转移瘤的PD-L1表达率为40.00%-66.67%，TPS评分与肺内肿瘤无显著差异。组织学和样本类型分析显示，腺癌类型和手术切除的肺外样本PD-L1表达评分显著高于同类型肺内肿瘤。临床病理参数分析显示，PD-L1阳性表达和高表达均与患者男性、吸烟史以及表皮生长因子受体（epidermal growth factor receptor, EGFR）野生型显著相关。

**结论:**

肺外转移性NSCLC样本的PD-L1表达评分高于肺内肿瘤，且不同部位组织样本的PD-L1表达阳性率存在差异。肺外转移性肿瘤与肺内肿瘤的PD-L1检测差异可能与不同转移部位、组织学和样本类型相关。

## Abstract (en)

**Background and objective:**

The expression of programmed cell death ligand 1 (PD-L1) as a biomarker for immunotherapy in non-small cell lung cancer (NSCLC) is routinely detected in clinical pathology department. However, the spatial heterogeneity of PD-L1 expression in intrapulmonary tumors and extrapulmonary metastases is still a challenge for the clinical testing. This study aims to explore the differences of PD-L1 expression in test samples obtaining from different sites of NSCLC. This study may contribute to the detection strategy of PD-L1 in patients with advanced lung cancer.

**Methods:**

One hundred and thirty-one cases of consecutively detected PD-L1 (22c3 assay, Dako) staining in metastatic NSCLC and 972 cases of non-paired intrapulmonary NSCLC were collected. The discrepancies of tumor proportion score (TPS) of PD-L1 expression in intrapulmonary samples and extrapulmonary metastatic samples of different sites were compared.

**Results:**

The positive expression rate of PD-L1 in extrapulmonary metastatic NSCLC (TPS ≥ 1%) was 61.83%, and the TPS was significantly higher than that in intrapulmonary tumors (*P*=0.03). The PD-L1 scores of the specimens obtained from different sites were significantly different (*P*=0.007). The positive rates of PD-L1 in liver and adrenal metastases were 85.71% and 77.78% respectively, and their TPS were significantly higher than that of the intrapulmonary samples (*P* < 0.05). The positive rates of PD-L1 in lymph node, bone, brain, soft tissue, and pleural metastases was 40.00%-66.67%, with no significant differences compared to intrapulmonary tumors. The analysis of histological subtype and sample type showed that the PD-L1 score of extrapulmonary samples of adenocarcinoma subtype or surgical specimen was significantly higher than that of intrapulmonary tumors. The analysis of clinicopathological parameters showed that the PD-L1 positive expression or high expression were significantly correlated with male patients, smoking history, and epidermal growth factor receptor (EGFR) wild type.

**Conclusion:**

The expression of PD-L1 in metastatic NSCLC is generally higher than that in intrapulmonary tumor, and the positive rate of PD-L1 expression was discrepant in different sites of specimen. The differences of PD-L1 score between extrapulmonary metastatic samples and intrapulmonary samples may be associated with different metastatic sites, histological subtype, and specimen type.

近年来，基于程序性死亡受体1（programmed cell death 1, PD-1）/程序性死亡配体1（programmed cell death ligand 1, PD-L1）的免疫检查点抑制剂（immune checkpoint inhibitors, ICI）的免疫治疗为晚期非小细胞肺癌（non-small cell lung cancer, NSCLC）患者带来了新的希望^[1]^。PD-L1作为肺癌免疫治疗的预测生物标志物，其临床应用价值在近年的大量临床研究中被证实，并被美国国立综合癌症网络（National Comprehensive Cancer Network, NCCN）指南推荐作为NSCLC病理常规检测项目。通过NSCLC样本组织的免疫组织化学染色（immunohistochemistry, IHC）检测肿瘤细胞的PD-L1表达状态，即肿瘤阳性比例评分（tumor proportion score, TPS）可预测NSCLC免疫治疗药物的疗效，对筛选治疗获益人群具有重要临床意义^[2-5]^。

在临床实践中，用于PD-L1检测的样本主要依赖于临床实践操作获取的组织样本。晚期NSCLC常伴有多发性远处转移。除肺部原发的肿瘤外，来自转移部位的组织样本，如淋巴结、骨、肾上腺等肺外转移的样本也常用于PD-L1检测。多项研究^[6-8]^表明PD-L1的表达存在时间和空间的异质性，可在肿瘤复发转移进展过程中产生变化，从而为晚期肺癌患者PD-L1的精准检测和样本选择带来了挑战。取材于转移部位NSCLC组织样本的PD-L1评分是否与肺内肿瘤组织样本具有异质性差异仍是临床检测所关注的重点问题^[9]^。由于临床实践中通常难以同时获取同一患者的多发转移瘤样本，因此PD-L1不同瘤灶间空间异质性的直接对比研究缺乏较大样本量的研究结果。目前国内仍缺乏相关临床研究数据表明不同部位NSCLC组织样本的PD-L1表达检测结果差异以及对免疫治疗获益人群预测的影响。

在本研究中，我们通过收集临床病理实际检测的病例，对NSCLC不同转移部位样本的PD-L1表达水平检测结果和同期的肺内肿瘤PD-L1表达的检测结果进行对比研究，探讨肺外样本与肺内样本的PD-L1评分差异以及转移部位对肿瘤PD-L1表达空间异质性的潜在影响，这对晚期肺癌患者的PD-L1检测策略具有重要帮助。

## 1 资料和方法

### 1.1 病例收集

研究回顾性收集了2020年6月-2021年4月期间北京大学肿瘤医院和天津医科大学肿瘤医院病理科PD-L1（22c3抗体）连续的临床实际检测的病例，均为初治患者并排除曾行新辅助治疗或既往肺癌治疗史的病例。最终纳入分析的病例为共计1, 103例，包含629例活检以及474例手术样本的病理资料；其中转移性肿瘤131例，同期非配对肺内肿瘤972例。131例转移性NSCLC中，转移部位包括淋巴结60例（局部转移12例和远处转移48例），脑16例、骨23例、胸膜10例、其他胸外实体器官16例（肾上腺9例、肝7例）、软组织6例。研究收集了组织病理蜡块的PD-L1（22c3抗体）临床病理科常规检测的切片，同时研究收集了临床病理特征资料包括性别、年龄、病理类型、吸烟状况以及已行检测的肺癌驱动基因表皮生长因子受体（epidermal growth factor receptor, *EGFR*）突变状况等。本研究由北京大学肿瘤医院伦理委员会（编号2018KT94）和天津市肿瘤医院伦理委员会（编号Ek2020140）批准。

### 1.2 PD-L1染色和判读

在本研究中所收集的PD-L1切片均是使用PD-L1 22c3抗体试剂盒（Dako, Carpinteria, CA, USA）和相应的Dako检测平台进行的标准化染色。根据PD-L1 22c3抗体试剂盒设定的标准化程序，使用Dako-Link AS-48自动免疫组化仪器对4 μm肿瘤组织切片进行免疫组织化学染色。PD-L1 22c3抗体染色的结果判读使用TPS，即阳性肿瘤细胞数占肿瘤细胞总数的比例×100%。由于所收集的PD-L1评分检测结果均是由受过专门培训的医师所签发，因此，本研究中TPS评分采用临床实际检测报告结果。根据目前对PD-L1 22c3抗体的临床应用指南，当TPS≥1%时视为PD-L1阳性表达；TPS为1%-49%时视为PD-L1低表达；TPS≥50%时视为PD-L1高表达。

### 1.3 统计分析

当PD-L1作为一个连续变量进行分析时，组间比较采用独立样本*t*检验比较组间差异，并使用*Kruskal-Wallis*检验进行多组间的差异比较。采用卡方检验和*Logistic*回归分析临床病理参数与PD-L1表达分类变量之间的关系。使用SPSS统计软件22版（IBM Corp., Armonk, NY）进行统计分析。*P* < 0.05为差异具有统计学意义。

## 2 结果

### 2.1 病例资料

在131例转移性NSCLC中，男性72例（55.0%），女性59例（45.0%）；平均年龄59.93岁，范围33岁-82岁。转移性肺腺癌111例（84.7%），鳞状细胞癌16例（12.2%），非特殊型或其他少见类型4例（3.1%）。在79例临床已检测EGFR的病例中，42例（53.2%）具有*EGFR*突变。同期972例肺内肿瘤中，男性537例（55.2%），女性435例（44.8%），平均年龄62.34岁，范围26岁-85岁，在450例临床已检测EGFR的病例中，243例（54.0%）具有*EGFR*突变。腺癌766例（78.8%），鳞状细胞癌177例（18.2%），非特殊型或其他少见类型29例（3.0%）。

### 2.2 肺外转移样本和肺内样本的PD-L1检测结果差异

肺外转移样本中，PD-L1表达的TPS评分平均值为26.24%，PD-L1阳性表达率为61.83%（81/131），高表达率为26.72%（35/131）；而肺内样本，TPS评分平均值为17.87%，阳性表达率为53.50%（520/972），高表达率为17.90%（174/972）。肺外样本的TPS评分显著高于肺内样本（*P*=0.03）。

研究比较了不同转移部位样本的PD-L1的阳性率差异。淋巴结转移样本PD-L1阳性率为60.00%（36/60），其中低表达率为33.33%（20/60），高表达率为26.67%（16/60）。其中，局部区域淋巴结转移样本PD-L1阳性表达率为58.33%（7/12），与远处淋巴结转移灶（60.42%, 29/48）相当。肺外实质器官转移样本中，肝转移样本PD-L1阳性表达率最高（85.71%, 6/7），其次为肾上腺转移样本（77.78%, 7/9）。软组织转移样本PD-L1阳性表达率为66.67%（4/6），脑转移瘤为62.50%（10/16），骨转移瘤为60.87%（14/23）；而胸膜转移瘤阳性表达率较低（40.00%, 4/10）（表 1，图 1A）。

**表 1:** NSCLC原发肿瘤和不同部位转移性肿瘤的PD-L1表达状况 PD-L1 expression in primary and metastatic NSCLC

Sites	*n*	TPS < 1%	TPS 1%-49%	TPS≥50%	TPS≥1%	PD-L1 score (average)
Primary NSCLC	972	452 (46.50%)	346 (35.60%)	174 (17.90%)	520 (53.50%)	17.87%
Metastatic NSCLC	131	50 (38.17%)	46 (35.11%)	35 (26.72%)	81 (61.83%)	26.24%
LN metastases	60	24 (40.00%)	20 (33.33%)	16 (26.67%)	36 (60.00%)	24.22%
Local LN metastases	12	5 (41.67%)	6 (50.00%)	1 (8.33%)	7 (58.33%)	10.75%
Distant LN metastases	48	19 (39.58%)	14 (29.17%)	15 (31.25%)	29 (60.42%)	27.58%
Liver metastases	7	1 (14.29%)	1 (14.29%)	5 (71.43%)	6 (85.71%)	61.71%
Adrenal metastases	9	2 (22.22%)	3 (33.33%)	4 (44.44%)	7 (77.78%)	47.78%
Soft tissue metastases	6	2 (33.33%)	2 (33.33%)	2 (33.33%)	4 (66.67%)	30.83%
Brain metastases	16	6 (37.50%)	6 (37.50%)	4 (25.00%)	10 (62.50%)	28.13%
Bone metastases	23	9 (39.13%)	11 (47.83%)	3 (13.04%)	14 (60.87%)	16.96%
Pleura metastases	10	6 (60.00%)	3 (30.00%)	1 (10.00%)	4 (40.00%)	9.70%
NSCLC: non-small cell lung cancer; PD-L1: programmed cell death ligand 1; TPS: tumor proportion score; LN: lymph node; TPS: tumor proportion score.						

**图 1:**
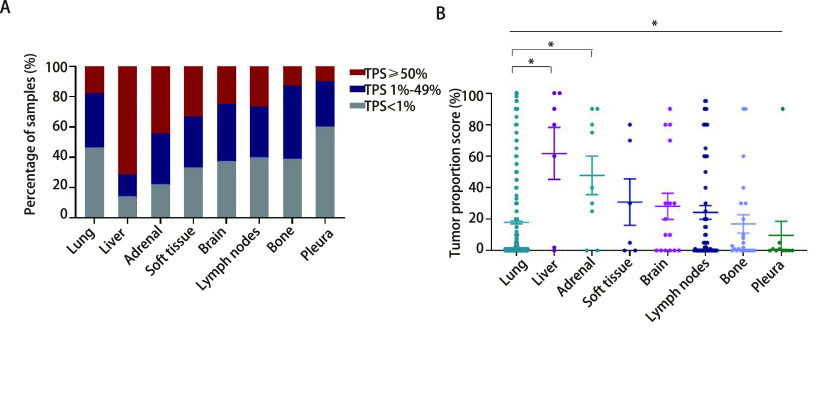
NSCLC不同部位样本的PD-L1阳性表达率（A）和肿瘤阳性比例评分差异（B） The discrepancies of PD-L1 positive expression (A) and tumor proportion score (B) in different sites of NSCLC

进一步比较不同部位样本的PD-L1评分差异，显示不同部位样本中TPS检测结果具有显著差异（*P*=0.007，*Kruskal-Wallis*检验）。肝脏转移样本的平均TPS评分最高，为61.71%，其次肾上腺转移样本为47.78%，两者均显著高于肺内样本的TPS评分（17.87%）（分别为*P* < 0.001、*P*=0.002，独立样本*t*检验）。软组织、脑、淋巴结转移样本的TPS评分分别为30.83%、28.13%和24.22%，均略高于同期肺内肿瘤，而骨转移和胸膜转移样本的TPS评分较低，分别为16.96%和9.70%，统计显示这些转移部位的TPS评分与肺内样本相比均无显著差异（表 1，图 1B）。

### 2.3 肺外转移样本和肺内样本不同组织学类型的PD-L1表达比较

111例肺外腺癌样本中，TPS评分平均值为27.14%，PD-L1阳性表达率为62.16%（69/111），高表达率为27.93%（31/111）；766例肺内腺癌样本中，TPS评分平均值为15.75%，PD-L1阳性表达率为49.22%（377/766），高表达率为15.80%（121/766）。两者比较显示，肺外腺癌样本的TPS评分显著高于肺内腺癌样本（*P* < 0.001）。

16例肺外鳞状细胞癌样本中，TPS评分平均值为25.00%，PD-L1阳性表达率为62.50%（10/16），高表达率为25.00%（4/16）；177例肺内鳞状细胞癌样本中，TPS评分平均值为25.15%，PD-L1阳性表达率为70.06%（124/177），高表达率为25.99%（46/177）；肺外鳞状细胞癌样本的TPS评分与肺内样本无显著差异（*P*=0.986）（图 2A）。

**图 2:**
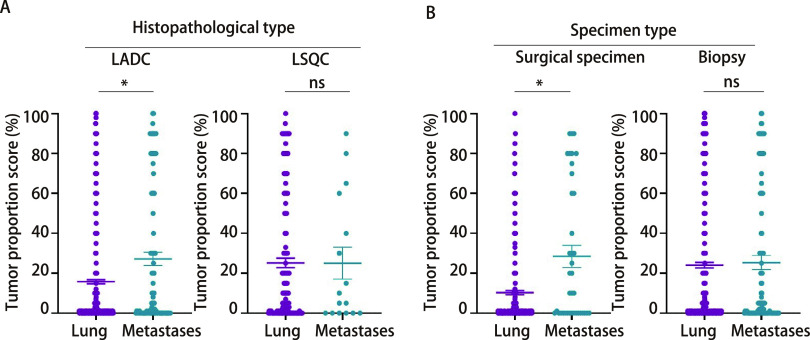
NSCLC肺外和肺内肿瘤中不同组织学类型和检测样本类型的PD-L1表达比较 The comparison of PD-L1 expression of extrapulmonary and intrapulmonary tumors in different histological subtype and specimen type of NSCLC. LADC: lung adenocarcinoma; LSQC: lung squamous carcinoma; ns: no significance.

### 2.4 肺外转移和肺内肿瘤不同样本类型的PD-L1表达比较

在38例转移肿瘤的手术样本中，TPS评分平均值为28.45%，PD-L1阳性表达率为57.89%（22/38），高表达率为28.95%（11/38）；在436例肺内手术样本中，PD-L1表达TPS评分平均值为10.27%，PD-L1阳性表达率为44.04%（192/436），高表达率为9.17%（40/436）。肺外转移与肺内肿瘤手术样本的TPS评分具有显著差异（*P* < 0.001）。

在93例转移肿瘤的活检样本中，TPS评分平均值为25.33%，PD-L1阳性表达率为63.44%（59/93），高表达率为25.81%（24/93）；在536例肺内肿瘤活检样本中，TPS评分平均值为24.05%，PD-L1阳性表达率为61.19%（328/536），高表达率为25.00%（134/536）。肺外转移与肺内肿瘤活检样本的TPS评分无显著差异（*P*=0.731）（图 2B）。

### 2.5 PD-L1表达与临床病理特征的相关性

在1, 103例NSCLC中，单因素分析显示PD-L1的阳性表达和高表达均与患者男性、吸烟史、组织学鳞状细胞癌类型以及*EGFR*野生型显著相关（均*P* < 0.05）；*Logistic*回归分析表明患者男性、具有吸烟史以及EGFR野生型均是PD-L1阳性表达的独立相关因素（均*P* < 0.05）（表 2）。

**表 2:** NSCLCPD-L1表达的临床病理特征 Clinicopathological correlations of PD-L1 expression in NSCLC

Variables	TPS < 1%	TPS≥1%	Univariate analysis	Multivariate analysis	TPS < 50%	TPS≥50%	Univariate analysis	Multivariate analysis
Gender			*P* < 0.001	*P*=0.005			*P* < 0.001	*P*=0.002
Male (*n*=609)	235	374			456	153		
Female (*n*=494)	267	227			438	56		
Age (yr)			*P*=0.718				*P*=0.578	
< 60 (*n*=393)	176	217			322	71		
≥60 (*n*=710)	326	384			572	138		
Smoker			*P* < 0.001	*P*=0.016			*P* < 0.001	*P*=0.048
Yes (*n*=601)	245	356			456	145		
No (*n*=502)	257	245			438	64		
Histological type			*P* < 0.001	*P*=0.269			*P*=0.006	*P*=0.680
LADC (*n*=877)	431	446			725	152		
LSQC (*n*=193)	59	134			143	50		
EGFR status			*P* < 0.001	*P* < 0.001			*P* < 0.001	*P* < 0.001
Mutant type (*n*=285)	172	113			265	20		
Wild type (*n*=244)	105	139			193	51		

在肺内肿瘤中，PD-L1的阳性表达和高表达均与患者男性、肿瘤组织类型显著相关（均*P* < 0.05），并与*EGFR*突变负相关（*P* < 0.001）。转移性NSCLC中，PD-L1的阳性表达和高表达与患者性别、年龄、吸烟史以及肿瘤组织类型无显著相关性（均*P* > 0.05）；PD-L1的高表达与*EGFR*突变呈显著负相关（*P*=0.008）（表 3）。

**表 3:** 转移性NSCLCPD-L1表达的临床病理特征 Clinicopathological correlations of PD-L1 expression in metastatic NSCLC

Variables	TPS < 1%	TPS≥1%	*χ* ^2^	*P*	TPS < 50%	TPS≥50%	*χ* ^2^	*P*
Gender			0.804	0.370			3.574	0.059
Male (*n*=72)	25	47			48	24		
Female (*n*=59)	25	34			48	11		
Age (yr)			0.008	0.929			0.24	0.624
< 60 (*n*=57)	22	35			43	14		
≥60 (*n*=74)	28	46			53	21		
Smoker			0.471	0.493			1.442	0.230
Yes (*n*=71)	29	42			49	22		
No (*n*=60)	21	39			47	13		
Histological type			0.001	0.979			0.060	0.806
LADC (*n*=111)	42	69			80	31		
LSQC (*n*=16)	6	10			12	4		
EGFR status			0.206	0.650			6.948	0.008
Mutant type (*n*=42)	18	24			36	6		
Wild type (*n*=37)	14	23			22	15		

### 2.6 *EGFR*主要突变位点与PD-L1表达的关系

285例*EGFR*突变病例中，PD-L1表达的TPS评分平均值为8.02%，PD-L1阳性表达率为39.65%（113/285），高表达率为7.02%（20/285）；EGFR野生型病例的TPS评分平均值为20.62%，PD-L1阳性表达率为56.97%（139/244），高表达率为20.90%（51/244）。EGFR野生型样本的TPS评分显著高于*EGFR*突变型（*P* < 0.001）。

*EGFR*突变位点检测结果显示，外显子21点突变（L858R）为158例（55.44%）、外显子19删失（Del 19）为102例（35.79%）以及其他少见位点的突变为25例（8.77%）。组间比较显示，不同*EGFR*突变位点之间TPS评分检测结果无显著差异（*P*=0.398）（图 3，表 4）。

**图 3:**
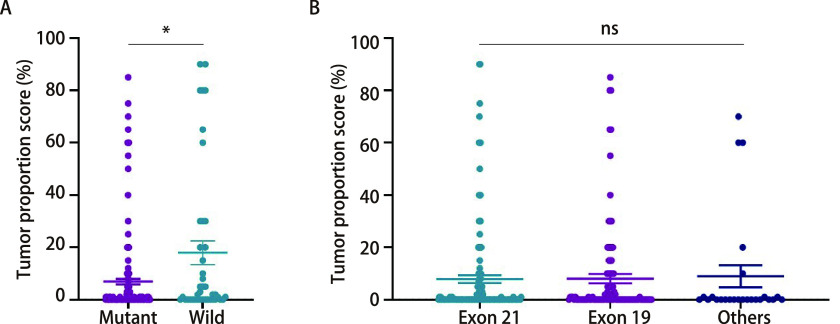
NSCLC中*EGFR*突变状态和不同突变位点中PD-L1表达的差异 Differences of PD-L1 expression according *EGFR* mutation status and mutation sites in NSCLC. EGFR: epidermal growth factor receptor.

**表 4:** NSCLC*EGFR*突变位点与PD-L1表达的关系 Relationship between *EGFR* mutation site and PD-L1 expression in NSCLC

EGFR status	Total cases	TPS≥1%	TPS≥50%
Wild type	244 (46.12%)	139 (56.97%)	51 (20.90%)
Mutant type	285 (53.88%)	113 (39.65%)	20 (7.02%)
Exon 21 (L858R)	158 (55.44%)	51 (32.28%)	11 (6.96%)
Exon 19 (Del 19)	102 (35.79%)	47 (46.08%)	6 (5.88%)
Others (Exon 20, 18, 14 *et al*)	25 (8.77%)	9 (36.00%)	3 (12.00%)

## 3 讨论

晚期NSCLC患者常表现为肺外转移，来自不同取样部位的肿瘤组织样本中PD-L1的表达差异是困扰临床检测的难题。在本研究中，我们对比取材于不同部位NSCLC样本的PD-L1检测结果，显示PD-L1表达水平在转移样本中的TPS评分显著高于肺内样本，且不同部位转移灶样本的PD-L1染色阳性率和TPS评分具有明显差异，表明PD-L1表达在肺外转移样本可存在异质性差异。

在转移性NSCLC中，多项研究^[10-12]^发现转移灶的PD-L1评分与原发灶存在不一致的现象。我们前期在NSCLC配对手术样本中对PD-L1表达水平定量分析显示，肿瘤转移灶相对于配对原发灶TPS评分明显升高^[13]^。在本研究队列中，PD-L1在实体器官肝脏和肾上腺转移灶中具有明显较高的阳性率，而在骨和胸膜转移瘤中表达水平较低。与本研究类似，Hong等^[14]^发现PD-L1在肾上腺、肝脏和淋巴结转移的PD-L1表达较高，而在骨和脑转移中表达较低。研究^[15, 16]^表明肿瘤细胞PD-L1的表达可能与所在转移部位的免疫微环境有关。肝脏和肾上腺这类富于血供的器官更容易募集效应T细胞进行有效的肿瘤免疫监测，该部位转移瘤中表达免疫检测点负性调控因子PD-L1的肿瘤细胞更易存活，并产生免疫抑制效应。因此肝、肾上腺转移的肿瘤细胞更有可能阳性表达PD-L1；而骨、软组织中招募效应T细胞能力较弱，肿瘤细胞可在较低的免疫选择压力下存活，PD-L1表达对免疫逃逸的作用较小，因此骨、胸膜等转移灶中PD-L1表达率较低。这表明PD-L1的表达调控随着肿瘤所在部位的不同可存在差异，可能是形成空间异质性的重要原因^[17]^。

不同转移部位的PD-L1评分可能对NSCLC中ICIs获益具有不同的预测价值。Qiao等^[18]^的研究显示肺癌肝转移患者无进展生存期（progression-free survival, PFS）最短，而基于ICI的联合治疗可以有效控制肝转移，延长PFS并改善客观缓解率（objective response rate, ORR）。Hong等^[14]^研究显示肺或远处器官转移样本中PD-L1水平越高，其应答率越高，而在淋巴结转移瘤中PD-L1评分和ICI的临床获益之间缺乏相关性。淋巴结组织对转移瘤的PD-L1调控可能由于其独特的免疫微环境而产生的免疫效应不同^[14]^。脑转移是晚期肺癌常见的远处转移部位。Mansfeld等^[6]^发现与配对的原发性肺癌样本相比，脑转移瘤中表达PD-L1较低。Kluger等^[19]^研究同样显示脑转移瘤中PD-L1的表达水平较低，可能与脑器官的免疫保护特征有关。但我们在本研究的脑转移灶中观察到PD-L1的表达并无明显下降趋势，而与肺内肿瘤阳性率相似，提示免疫治疗应用于脑转移瘤的潜力。

目前，NSCLC中不同部位组织样本产生PD-L1表达空间异质性的相关因素和机制仍然不明。本研究显示转移肿瘤和肺内肿瘤的PD-L1表达评分差异主要体现于肺腺癌中，而在肺鳞状细胞癌类型中无显著差异，提示肿瘤PD-L1的空间异质性存在组织学类型差异。考虑到活检样本和手术样本之间潜在的异质性，研究对比了两种类型的样本对PD-L1表达水平差异的影响，结果显示肺外和肺内肿瘤活检样本的PD-L1表达阳性率无明显差异，而手术样本之间的PD-L1表达差异明显，提示用于检测的样本类型对PD-L1检测结果具有潜在影响。

通过对本研究NSCLC病例的PD-L1表达评分与临床病理特征参数进行相关性分析，发现PD-L1的阳性表达与患者男性、吸烟史、组织类型以及*EGFR*野生型显著相关，这与我们前期的研究以及多个亚洲人群的研究相似^[20-23]^。这些临床基线特征与PD-L1的表达密切相关，是否对PD-L1评分的异质性产生显著影响需要较大样本的配对病例进行分析。有研究^[24]^显示NSCLC中EGFR信号通路的激活抑制PD-L1和上调免疫抑制因子的表达，从而促进肿瘤免疫微环境的免疫抑制作用。本研究分析显示，转移瘤中PD-L1的高表达与*EGFR*突变呈显著负相关，但不同*EGFR*的突变位点的患者PD-L1表达阳性率并无明显差异。

PD-L1表达的空间异质性也可表现为同一患者不同转移瘤灶间的不同，这提示活检取样部位的不同可导致PD-L1表达检测结果的不同。因此，在具有远处转移的晚期NSCLC患者进行免疫治疗的过程中，应考虑到PD-L1在肺外转移灶和肺内样本之间的异质性变化。同一患者中不同转移瘤灶间的PD-L1表达差异是否对各个瘤灶的免疫治疗效果产生影响仍然需要进一步研究探讨。由于临床上很难获取同时具有多发转移瘤的组织样本，实际研究具有一定的困难。

作为一个临床实践样本数据的回顾性分析研究，本研究个别部位的转移样本，如肝转移、软组织转移等病例数较少。在后续的临床研究需要进一步的大样本量的队列研究，并且探讨不同部位PD-L1表达水平与肺癌免疫治疗疗效及预后的相关性。此外，目前TPS是PD-L1抗体22c3的NCCN指南推荐的检测指标，其在免疫细胞评分的异质性差异，仍需要进一步探索。

综上所述，本研究表明NSCLC转移样本中PD-L1表达水平通常要高于肺内肿瘤。不同的转移部位样本的PD-L1评分具有差异，其中肝脏和肾上腺阳性率较高、骨和胸膜转移灶阳性率较低。肺外和肺内样本的PD-L1检测结果差异可能与组织学类型和取材样本类型有关。探讨不同转移部位对PD-L1检测结果的影响，有助于理解PD-L1表达的空间异质性，并对指导晚期多发转移的肺癌的PD-L1的检测策略具有一定帮助。
